# Proximal hyperspectral detection of rice and weed: characterization and discriminant analysis

**DOI:** 10.3389/fpls.2025.1685985

**Published:** 2025-12-11

**Authors:** Zhentao Wang, Tenghui Lin, Huijie Li, Yanling Yin, YuTing Suo, Hongfei Yang, Yulin Li, Fengjie Cai, Li Xiao

**Affiliations:** 1 College of Mechanical and Electrical Engineering, Shihezi University, Shihezi, China; 2 Key Laboratory of Northwest Agricultural Equipment, Ministry of Agriculture and Rural Affairs, Shihezi University, Shihezi, China; 3 Xinjiang Production and Construction Corps Mechanization Engineering Laboratory for Special Crop Production, Shihezi University, Shihezi, China; 4 College of Engineering, Northeast Agricultural University, Harbin, China; 5 College of Medicine, Shihezi University, Shihezi, China; 6 State Key Laboratory for Crop Stress Resistance and High-Efficiency Production, Shaanxi Key Laboratory of Agricultural and Environmental Microbiology, College of Life Sciences, Northwest A&F University, Yangling, Shaanxi, China; 7 Yantai Agricultural Technology Popularization Center, Yantai, China

**Keywords:** rice, weed, hyperspectral imaging technology, deep learning, identification

## Abstract

**Introduction:**

Weeds represent a critical component of agricultural biodiversity and contribute to a range of ecosystem services, yet they remain a major constraint on global crop production. Remote sensing technology, particularly hyperspectral imaging, has advanced from spectral response patterns to species identification and vegetation monitoring. Consequently, the ability to accurately map weed species and assess their physiological activity in agricultural settings is of growing important.

**Methods:**

In this study, we established a hyperspectral library of rice and weed species in cold regions of northern China, comprising a total of 36 species. Using a ground-based hyperspectral camera (SPECIM-IQ), we collected 1080 hyperspectral images and extracted representative spectral reflectance curves for rice and 35 weed species. We employed canopy spectral profile characteristics, vegetation indices, and principal component analysis (PCA) to characterize and explain the differences among various weeds.

**Results:**

A novel deep learning network, SS-CNN, was developed to identify rice and weed species from hyperspectral imagery, and ablation experiments were conducted to evaluate its performance. When the training sample size (Tr) was set at 70%, the SS-CNN model outperformed the comparative models with the best identification results (overall accuracy (OA): 99.910%, average accuracy (AA): 99.502%, Kappa: 0.9991). Even at a reduced training sample size of 5%, the SS- CNN algorithm maintained optimal classification performance (OA: 95.370%; AA: 86.468%; Kappa: 0.9518).

**Discussion:**

This study demonstrates the application of proximal hyperspectral remote sensing and deep learning networks for rice and weed identification and characterization in harsh field scenarios. It provides a valuable baseline for understanding the hyperspectral characteristics of paddy field weed stress and monitoring their growth status.

## 1 Introduction

Weeds play a crucial role in enriching biodiversity and ecosystem services, such as promoting biological evolution, improving ecological environments, and maintaining climate stability (Korres et al., 2016; Guo et al., 2018; Gage and Schwartz-Lazaro, 2019). Maintaining a certain level of weed diversity in farmlands also benefits nutrient cycling, protecting natural enemies of pests, and halting soil erosion (Librán-Embid et al., 2020; Quan et al., 2023). An important constraint on rice yield and quality is weed competition for natural resources, which can cause crop growth disruptions, changes in external morphological characteristics, biochemical component content, photosynthetic mechanism efficiency, chlorophyll, and other pigments in rice plants, and, in extreme cases, crop failure (Zhang et al., 2019; Sulaiman et al., 2022). Furthermore, the extent of these adverse effects often varies with weed species, their growth habits, and environmental conditions (Malik and Singh, 1995; Dass et al., 2017). Therefore, in order to promptly estimate the heterogeneity, weed stress and mitigate adverse effects on crop productivity and the environment, the ability to create weed species maps for agricultural fields and analyze weed physiological activity has become of paramount importance. However, weed information collection and regular updates of biophysical conditions for weed data are essential for tracking weed status, which in turn poses challenges to current weed physiological activity monitoring and species identification using traditional visual weed assessment (Chen et al., 2013; Liu et al., 2022). Furthermore, traditional methods are associated with escalating costs, as they involve time-consuming, labor-intensive, and financially burdensome fieldwork. Consequently, there is an urgent demand for a reliable, efficient approach capable of detecting subtle variations in plant growth, physiological status, and productivity parameters that are specific to different plant species. Importantly, such an approach should also identify the key drivers of these variations, thereby facilitating the optimization of subsequent crop management strategies and resource allocation.

With the advancement of remote sensing technology, it has become possible to conduct large-scale, regular observations, create weed distribution maps, and monitor changes in agricultural environments using state-of-the-art satellite and aerial sensors. These satellite and aerial sensors offer a wide range of imagery, including RGB, multi-spectral and hyperspectral images. However, despite the recent launch of satellites providing sub-meter ground resolution (Congalton et al., 2014), the satellites’ altitude, weather conditions, and highly mixed farmland communities, as well as the Chinese BeiDou Navigation Satellite System (BDS), Geostationary Orbit (GEO), and Inclined Geostationary Orbit (IGSO), also have systematic errors in orbital angles related to solar radiation pressure (SRP) (Guo et al., 2016). These factors greatly affect the retrieval accuracy of aviation and aerospace images. This implies that, in many cases, detection is only possible when defects are already widespread. On the other hand, drones can offer ground sample distances (GSD) of less than 1 centimeter. However, this technology also comes with some drawbacks: operating drones requires specific training, flight regulations may be stringent, crashes are common, and weather conditions can reduce image quality or even impede flights (Barbedo, 2019). Additionally, due to the heterogeneity of agricultural systems, complex crop cycles, and the similarity in color and spectral characteristics between weeds and crops, weed and crop detection from hyperspectral images is a challenging task (Wang et al., 2022b; Coleman et al., 2023). The classification process becomes even more challenging in the presence of crop occlusion and complex background environments, such as soil. Therefore, the use of *in-situ* or ground-level spectroscopy for close-range image measurements is of-ten a more feasible option to achieve high spatial resolution.

Some hyperspectral databases composed of plant-specific information have been established, providing important support for the development of environmental protection, sustainable agriculture, and other fields. Laporte-Fauret et al (Laporte-Fauret et al., 2020). conducted a comprehensive multi-scale survey of coastal dune systems located approximately about 20 km southwest of France, and generated a spectral library of vegetation community ground cover types in dune areas to characterize the spatial distribution of stability patterns in coastal dunes. Prasad et al (Prasad et al., 2015). collected canopy-level field spectra and leaf-level laboratory spectra of 34 species from two different mangrove ecosystems on the eastern coast of India, and the removal of water vapor absorption bands and smoothing of spectra to develop an exclusive spectral library of mangrove species to generate species level mangrove mapping of other regions will be taken up in future studies. Chi et al (Chi et al., 2021). developed a spectral library of 16 common vegetation species and mosses in the Ant-arctic ice-free region using field spectrometers, and analyzed them using spectral dis-crimination measures to observe the spectral characteristics and potential separability of Antarctic plants in different wavelength ranges.Su et al (Su et al., 2022). analyzed (spectrally) and mapped the distribution of triticale weeds in wheat fields by integrating unmanned aerial vehicle (UAV), multispectral imagery, and machine learning techniques. Mkhize et al (Mkhize et al., 2024). recorded 165 GPS points of weed, maize and mixed categories in the early growth stages of six maize farms, these GPS points were superimposed on Sentinel-2 images within two days after obtaining field data to guide the collection of spectral features of maize, mixed and weed categories. Dai et al (Dai et al., 2023). collected 700 sets of spectral data of wheat and weeds, and the classical support vector machine (SVM) based on spectral data was applied to identify weeds to study the classification performance of wheat and weeds in different bands. While existing spectral libraries encompass a broad range of spectral information, they offer limited coverage of spectral characteristics for weed species in agricultural ecosystems, especially in paddy fields. Unique to paddy fields, species such as *E.crus-galli* and *S.trifolia*, are unlikely to be found from these spectral libraries. Northern Chinese cold paddy fields are renowned for their high weed species diversity and distinctive agricultural landscape features, with most weed growth occurring in the intense field environment characterized by a network of canals and vast fertile expanses. Therefore, this study takes the rich weed species in paddy fields in northern China as the research object, and pro-vides useful contributions to the development of hyperspectral images and databases to fill the gap in the study of weed stress species in paddy fields in northern China.

Considering the above background, the work conducted in this study is outlined as follows:(1) A hyperspectral library for rice fields in northern China was established. From May to September 2023, a total of 1080 high-spectral images were collected in various rice cultivation areas, covering 36 species (belonging to 18 families and 36 genera) of both rice and weeds. (2) The physiological activity of rice and weed was indirectly characterized using canopy spectral profile characteristics and vegetation indices. (3) A deep neural network was developed to identify rice and weed species in real agricultural field conditions. We also analyzed the results of model ablation experiments conducted under different modeling strategies, discussing the roles of different modules within the deep neural networkxa.

## 2 Materials and methods

### 2.1 Test sample acquisition

**Figure 1:**
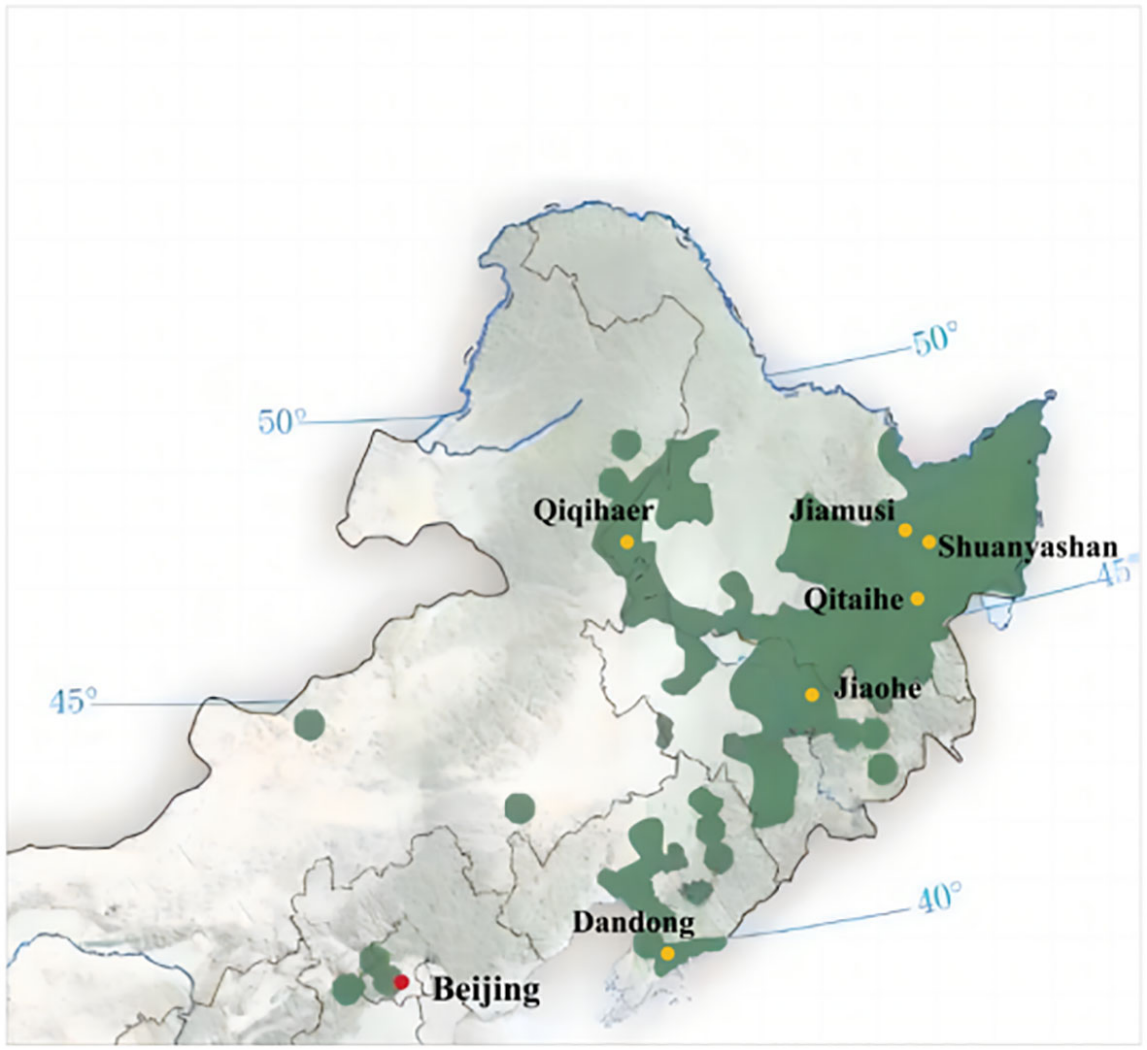
Rice planting areas (green areas) and sampling sites (yellow spots) in northern China.

**Figure 2:**
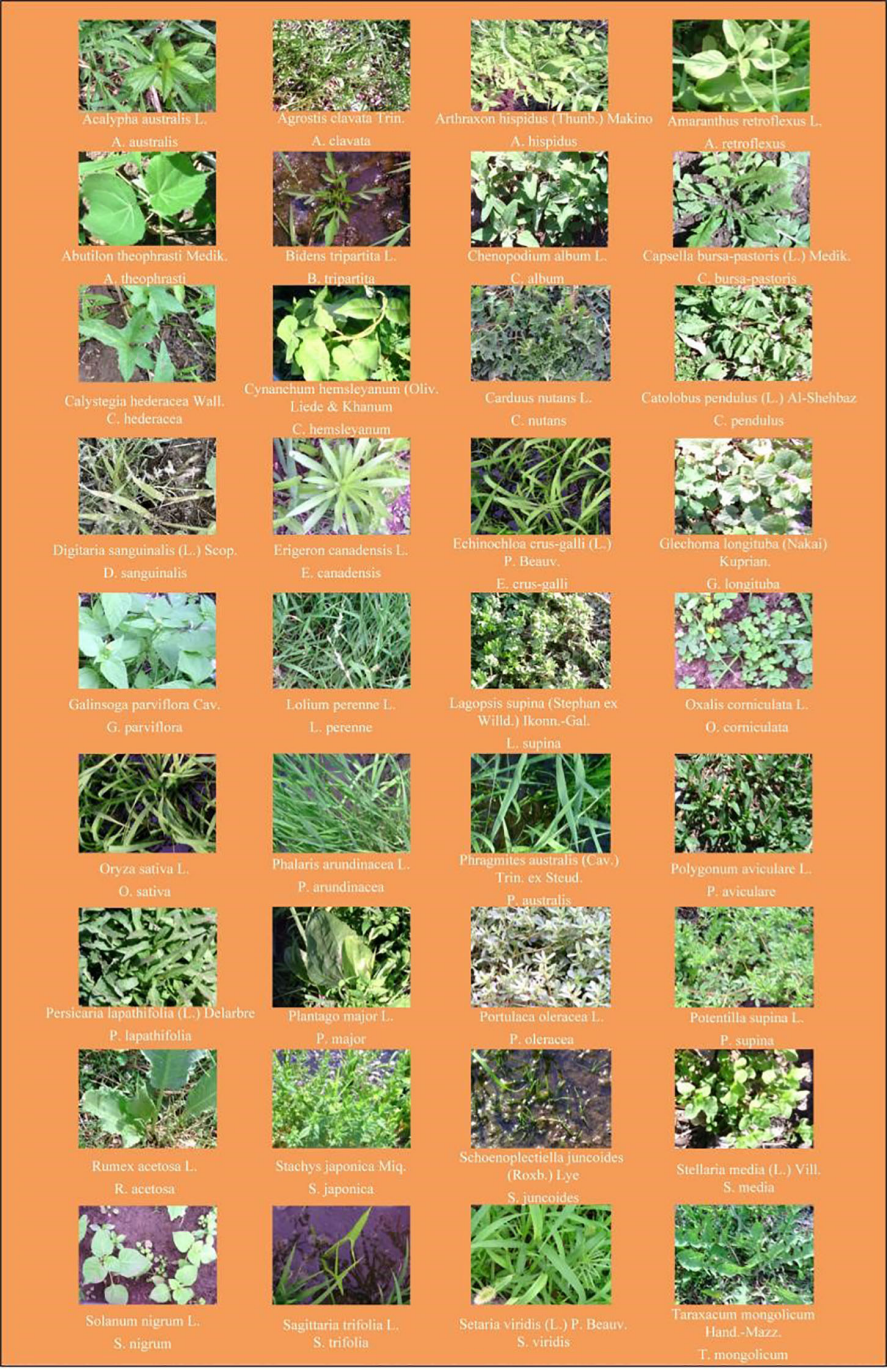
Photos, species and abbreviation of rice and weed samples.

Aromatic rice type 2 (*O. sativa*) with 13 leaves and 135 days of growth period, which is widely planted in the rice-growing areas of northern China, was selected for this study. The criteria for selecting weed species in paddy fields included but were not limited to factors such as weed species, size, spatial distribution within the field, proximity to field dikes, traffic flow on adjacent agricultural roads, and sample spatial distribution. The chosen weed species were collected from six different locations in the major rice-producing regions of northern China, as depicted in **Figure 1**. These sampling points encompassed (1) Dandong City (123°37′16.59″N ~ 125°10′24.77″N, 40°44′50.65″E ~ 41°5′12.16″E), (2) Jiaohe City (127°3′30.82″N ~ 128°1′33.04″N, 43°28′44.08″E ~ 44°9′58.18″E), (3) Qitaihe (130°13′22.97″N ~ 131°42′11.23″N, 45°42′41.36″E ~ 46°9′51.20″E), (4) Qiqihar City (123°22′59.83″N ~ 126°14′44.84″N, 46°19′56.73″E ~ 48°10′13.77″E), (5) Shuangyashan City (131°49′28.03″N ~ 134°7′27″N, 45°50′3.93″E ~ 47°28′29.04″E), and (6) Jiamusi City (129°42′51.22″N ~ 134°24′46.84″N, 46°33′5.85″E ~ 48°21′41.30″E). Detailed information regarding the weed species is provided in **Figure 2**, encompassing a total of 18 families, 35 genera, and 35 species.

### 2.2 Hyperspectral image acquisition and correction

**Figure 3:**
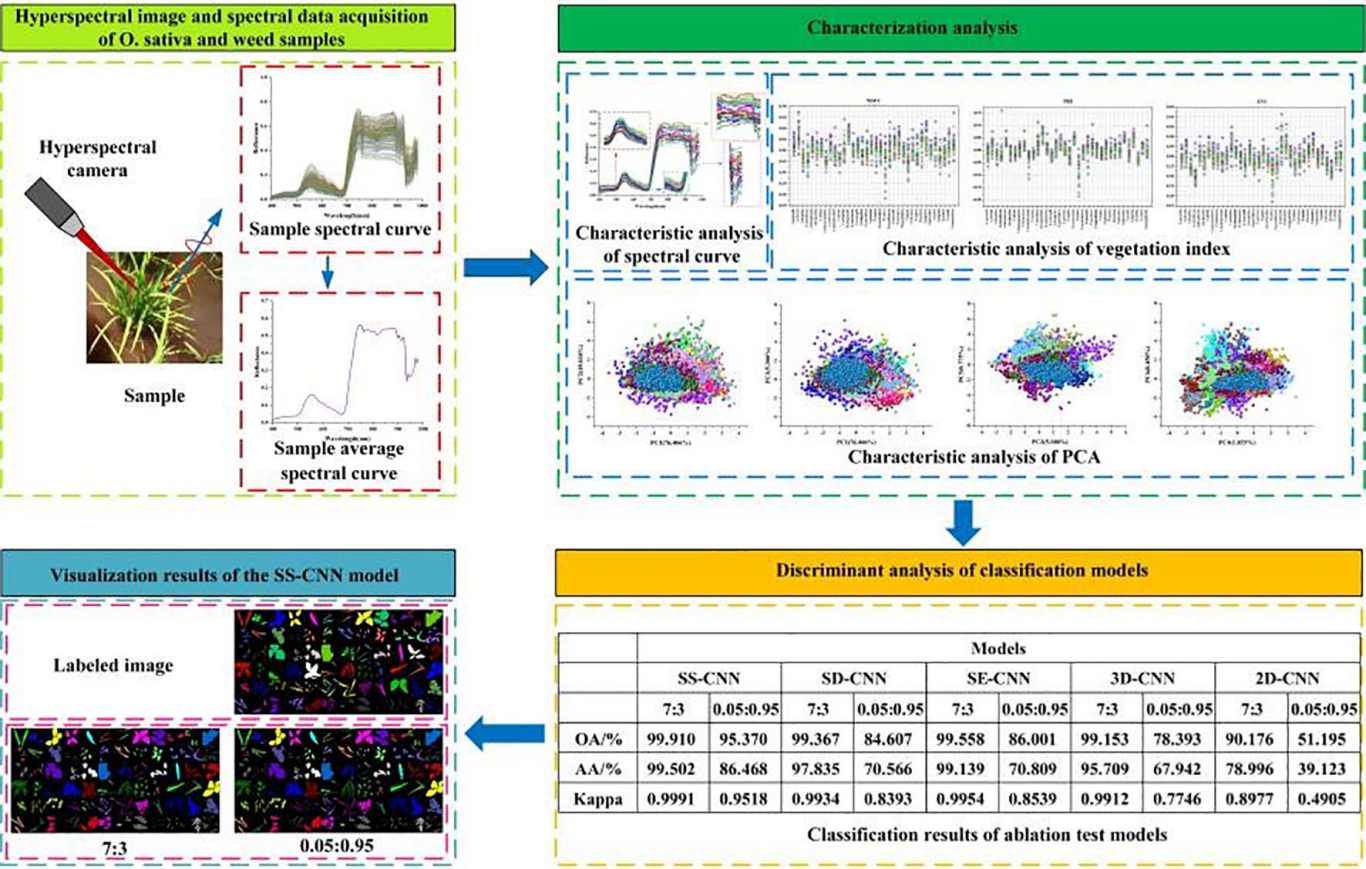
Hyperspectral image information acquisition flow chart.

In this study, a Proximal hyperspectral camera (Model SN: 190-1100381, SPECIM, Spectral Imaging Ltd., Oulu, Finland) was used to obtain images of rice and weed samples. The camera, with dimensions of 207×91×74 mm and a 12.5 mm lens, featured a total of 204 spectral bands covering a wavelength range from 397 to 1003 nm. The spectral resolution was 7 nm, and the images were captured at a resolution of 512×512 pixels. The laptop and hyperspectral camera were connected via a USB cable, and high-resolution hyperspectral images were acquired using Specim IQ Studio software. The process of hyperspectral image information acquisition is shown in **Figure 3**.

Before commencing the image acquisition experiments, the hyperspectral camera was preheated for 30 minutes. In order to ensure the accuracy of the data, this study used the Labsphere Spectralon panel for calibration. Since the Labsphere Spectralon panel (nominal reflectance of 99%) has almost perfect reflectance in the range of 250–2500 nm, it is used as a reference in field measurements. The camera’s and stand’s positions were adjusted to ensure that the samples fell within the camera’s scanned range. Due to atmospheric effects, the solar radiation changes in the process of returning from the ground object to the camera, leading to discrepancies between the recorded spectral information in hyperspectral images and the actual information (Zhao, 2004). To ensure consistency between the information captured in the field and the actual data in the acquired hyperspectral images, the Quick Atmospheric Correction (QUAC) algorithm was employed for atmospheric correction. This correction eliminates the effects of illumination as well as atmospheric components such as water vapor, oxygen, carbon dioxide, methane, and ozone on the reflectance of the ground objects, thus enabling the retrieval of the actual surface reflectance of ground objects (Bernstein et al., 2012). The QUAC algorithm calculates atmospheric transmittance and atmospheric reflectance based on the uncorrected image and the reflectance model. It then applies this information to correct the image, removing atmospheric influences.

In order to obtain the representative spectral information of the sample, refer to the research of Wang et al (Wang et al., 2022a). This study initially extracts a single-band image from the atmospherically corrected hyperspectral image, which serves as a grayscale image. Subsequently, the grayscale image undergoes mask processing to generate a binary image. Finally, individual rice or weed plants in the binary image are selected as regions of interest (ROI). The average spectral information extracted from these ROIs is considered as the hyperspectral data for the sample. Additionally, for each pixel, a reflectance transformation is applied using the Labsphere Spectralon panel as a reference region, following Equation 1.

(1) $$\text{Re}=\frac{{\text{D}}_{\text{Raw}}-{\text{D}}_{\text{Dark}}}{{\text{D}}_{\text{White}}-{\text{D}}_{\text{Dark}}}$$

where ${\text{R}}_{\text{e}}$is the reflectance data of a pixel; ${\text{D}}_{\text{Raw}}$ is the raw data of that pixel; ${\text{D}}_{\text{Dark}}$ is the current value taken from the dark frame; and ${\text{D}}_{\text{White}}$ is the average value of the selected white reference region.

### 2.3 Construction of deep neural network classification model for rice and weed species identification

#### 2.3.1 SS-CNN

**Figure 4:**
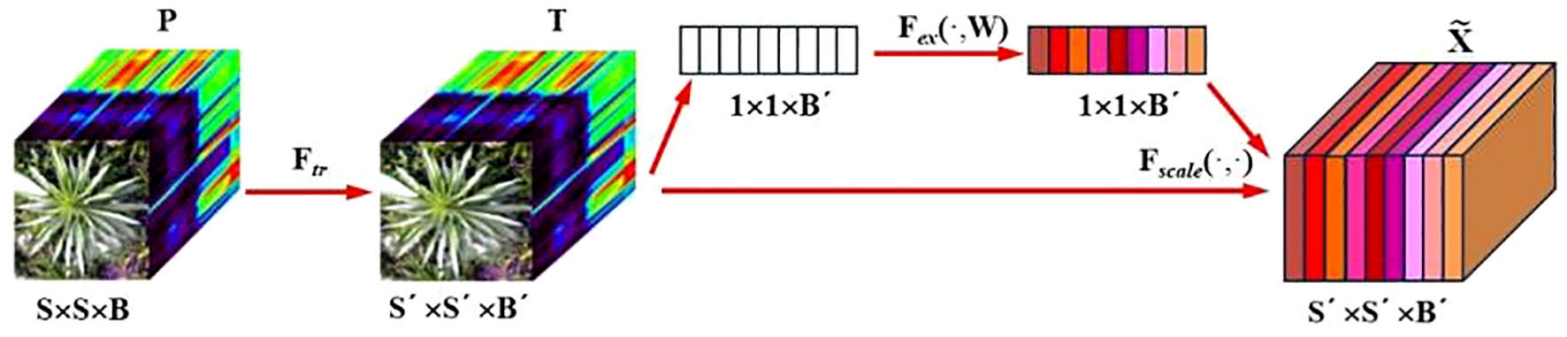
Squeeze-and-excitation networks.

A deep neural network framework was developed for training and validating species-level classification of rice and weed species based on hyperspectral image spatial-spectral features. Neural networks, inspired by the human brain, are a set of algorithms designed for pattern recognition. With the rapid advancement of computer hardware and the proliferation of data, neural networks have proven to be one of the most effective tools for data analysis (He et al., 2015). We established a deep neural network named SS-CNN (Superpixelwise Division-Squeeze-and-Excitation Convolutional Neural Network) for identifying 35 weed species and rice in paddy fields in northern China. The network’s architecture is illustrated in **Figure 4**. The network takes hyperspectral images as input and the probabilities of rice and weed species as output. It comprises an input layer, a superpixel segmentation module, three 3D convolutional layers (with 8, 16, and 32 neurons, respectively), two Batch Normalization (BN) layers, four 2D convolutional layers (with 32, 64, 128, and 256 neurons, respectively), an SE attention mechanism module, a pooling layer, a flatten layer, two fully connected layers, and an output layer with 36 neurons. The SoftMax function is applied at the output layer to generate probabilities.

After the hyperspectral image is input into the input layer, in order to make full use of the spatial information contained within the HSI cube, the superpixelwise division module is used to segment the hyperspectral image by using the entropy rate superpixel (ERS). Here, the original hyperspectral image (M is the height of the image, N is the width of the image, and C is the number of bands of the spectrum) is reduced by the superpixelwise division module. The size of the data cube U becomes MNB. The number of spectral bands is reduced from C to B, while maintaining the same spatial dimensions, namely, width M and height N. This operation retains crucial spatial information for object recognition while eliminating spectral redundancy. Subsequently, the HSI data cube processed by the superpixel segmentation module is divided into small overlapping 3D blocks to serve as input data for the model, with the true labels determined by the labels of the center pixels. We generate 3D neighboring patches, denoted as, from U with spatial location (α, β) as the center, covering a -window or spatial range and all B spectral bands.

To concurrently enhance the number of spectral-spatial feature maps, 3D convolutions are employed in triplicate, effectively preserving the spectral characteristics of the input hyperspectral (HSI) data within the output volume (He et al., 2023). The dimensions of the 3D convolution kernels are set to 3x3x7, 3x3x5, and 3x3x3, corresponding to two spatial dimensions and one spectral dimension. The number of convolution kernels is 8, 16, and 32, respectively. Furthermore, Batch Normalization (BN) layers are incorporated following the 3D convolution layers to process the extracted image features. Specifically, BN layers expedite network training and convergence while mitigating overfitting. Subsequently, 2D convolution layers are employed to extract spatial features, enabling a robust differentiation of spatial information within distinct spectral bands without significant loss of spectral information. The dimensions of the 2D convolution kernels are uniformly set to 3x3, encompassing two spatial dimensions, and the number of convolution kernels is 32, 64, 128, and 256.

As a measure to mitigate the issues of gradient vanishing or exploding, the present study employed the Softsign activation function. The Softsign activation function exhibits a gentler curve, and the derivative decreases slowly. This characteristic enables it to alleviate the gradient vanishing problem and facilitate more efficient learning (Wang et al., 2019). Its mathematical expression is represented as in Equation 2:

(2) $$\text{f}(\text{x})=\frac{\text{x}}{1+|\text{x}|}$$

Convolutional features introduce nonlinearity into the model through activation functions (Roy et al., 2019). In 3D convolutional layers, activation values at spatial position (x, y, z) of the *j*th feature map in the *i*th layer are computed using Equation 3 and denoted as ${\text{v}}_{\text{i},\text{j}}^{\text{x},\text{y},\text{z}}$.

(3) $${\text{v}}_{\text{i},\text{j}}^{\text{x},\text{y},\text{z}}=\phi ({\text{b}}_{\text{i},\text{j}}+\sum_{\text{τ}=1}^{{\text{d}}_{\text{l}-1}}\sum_{\text{λ}=-\text{η}}^{\text{η}}\sum_{\text{ρ}=-\text{γ}}^{\text{γ}}\sum_{\text{σ}=-\text{δ}}^{\text{δ}}{\text{ω}}_{\text{i},\text{j},\text{τ}}^{\text{σ},\text{ρ},\text{λ}}\times {\text{v}}_{\text{i}-1,\text{τ}}^{\text{x}+\text{σ},\text{y}+\text{ρ},\text{z}+\text{λ}}$$

where ϕ is the activation function, ${\text{b}}_{\text{i},\text{j}}$is the bias parameter for the *j*th feature map of the *i*th layer, ${\text{d}}_{\text{l}-1}$is the number of feature map in (l-1)th layer and the depth of kernel ${\text{ω}}_{\text{i},\text{j}}$for the jth feature map of the *i*th layer, 2η+1 is the depth of kernel along spectral dimension, 2γ+1is the width of kernel, 2δ+1is the height of kernel and ${\text{ω}}_{\text{i},\text{j}}$is the value of weight parameter for the *j*th feature map of the *i*th layer.

In the 2D convolution layer, the activation value at spatial position (x, y), in the *j*th feature map of the *i*th layer, denoted as ${\text{ v}}_{\text{i},\text{j}}^{\text{x},\text{y}}$, as shown in Equation 4:

(4) $${\text{v}}_{\text{i},\text{j}}^{\text{x},\text{y}}=\phi ({\text{b}}_{\text{i},\text{j}}+\sum_{\text{τ}=1}^{{\text{d}}_{\text{l}-1}}\sum_{\text{ρ}=-\text{γ}}^{\text{γ}}\sum_{\text{σ}=-\text{δ}}^{\text{δ}}{\text{ω}}_{\text{i},\text{j},\text{τ}}^{\text{σ},\text{ρ}}\times {\text{v}}_{\text{i}-1,\text{τ}}^{\text{x}+\text{σ},\text{y}+\text{ρ}}$$

**Figure 5:**
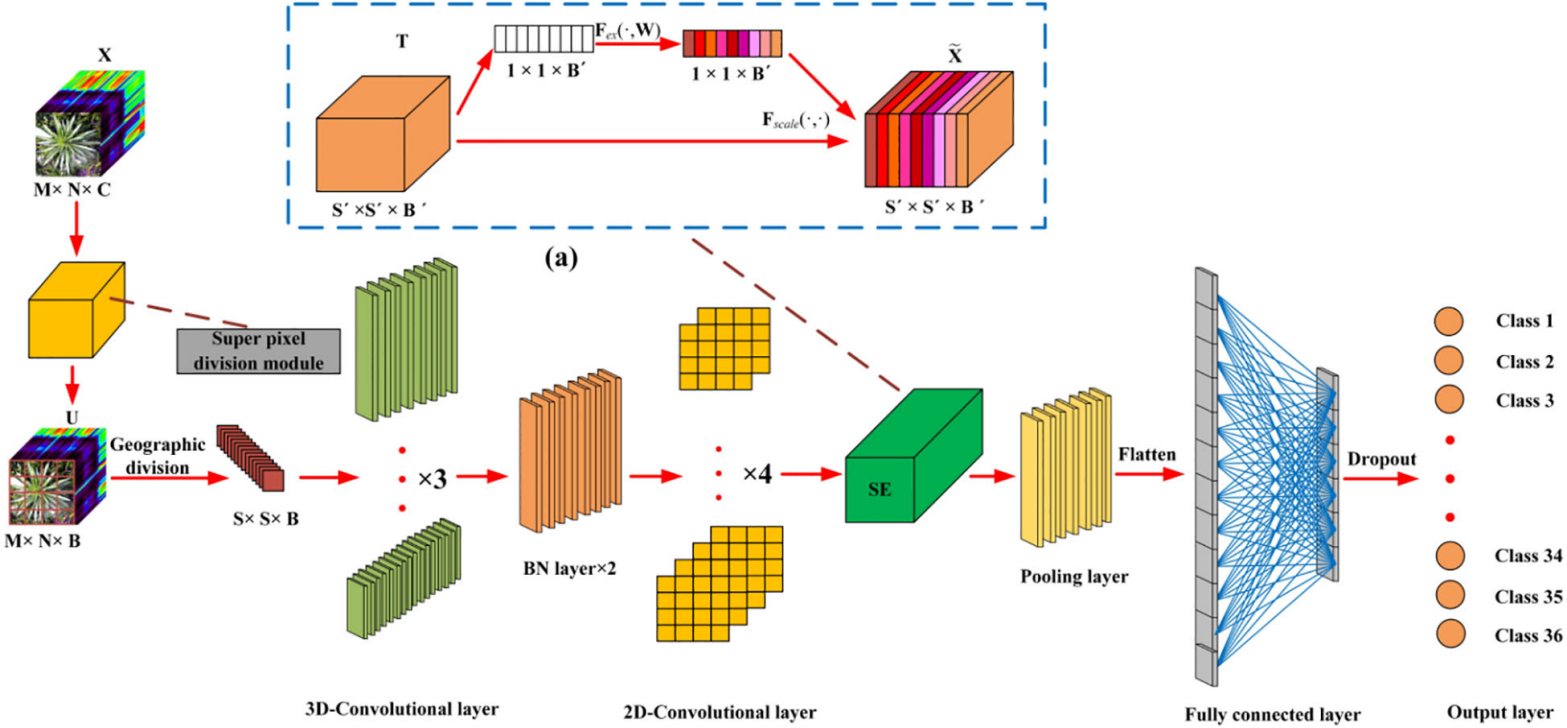
SS-CNN structure diagram.

The SE attention module (Squeeze-and-Excitation Networks) proposed by Hu et al (Hu et al., 2020). is introduced after the 3D-2D convolutional layer (**Figure 5**). Features P extracted by the convolution layers initially undergo a squeeze operation, which aggregates feature maps across the spatial dimensions a×a to generate channel descriptors. These descriptors embed the global distribution of channel feature responses, enabling information from the network’s global receptive field to be utilized by lower layers. Subsequently, an excitation operation follows, in which an auto-gating mechanism based on channel dependencies is employed to learn specific activations for each channel, controlling the excitations for each channel. The feature map P is then reweighted to produce the output of the SE block, which can be directly fed into the subsequent pooling layer.

In the final step, the processed feature maps are flattened into one-dimensional vectors using a ‘flatten’ layer and fed into fully connected layers for further abstract feature restructuring. This study incorporates two fully connected layers with 256 and 128 neurons, respectively. Furthermore, Dropout regularization is applied to prevent overfitting, with a parameter set to 0.4. The SS-CNN algorithm employs the backpropagation algorithm with random weight initialization and training. We employ mini-batches of size 32 and train the network for 50 epochs, without batch normalization and data augmentation. The optimal learning rate is chosen to be 0.0001.The optimizer is Adam, and the loss function is the category cross entropy loss. The layer wise summary of the proposed SS-CNNt architecture with window size 25×25 (**Table 1**).

**Table 1:** The layer wise summary of the proposed SS-CNN architecture with window size 25×25.

No.	Layer (type)	Kemel size	Output shape	Activation	Param
1	Input_1 (Input Layer)		(25, 25, 25, 1)	`	
2	Conv3D_1 (Conv3D)	(3, 3, 7)	(23, 23,19, 8)	ReLU	512
3	Conv3D_2 (Conv3D)	(3, 3, 5)	(21, 21, 15,16)	ReLU	5776
4	Conv3D_3 (Conv3D)	(3, 3, 3)	(19, 19, 13,32)	ReLU	13856
5	reshape_1 (Reshape)		(19, 19, 416)		
6	BN_1		(19,19,416)		
7	BN_2		(19,19,416)		
8	conv2d_1 (Conv2D)	(3,3)	(17, 17, 32)		119840
9	conv2d_2 (Conv2D)	(3,3)	(15, 15, 64)		10816
10	conv2d_3 (Conv2D)	(3,3)	(13, 13, 128)		15488
11	conv2d_4 (Conv2D)	(3,3)	(11, 11, 256)		20736
12	SE		(11, 11, 256)		64
13	Flatten_1 (Flatten)		2402816		
14	Dense_1 (Dense)		256	ReLU	18934190
15	Dropout_1 (Dropout)		256		
16	Dense_2 (Dense)		128	ReLU	526063
17	Dropout_2 (Dropout)		128		
18	Dense_3 (Dense)		2	Softmax	258

#### 2.3.2 Ablation experiments

In this study, we propose a Spectral-Spatial 3D-2D Hybrid Deep Neural Network (SS-CNN) based on hyperspectral images. It uses 3D-2DCNN as the feature extraction network, and superpixelwise division module and SE attention module as the enhanced spatial-spectral feature extraction module. To demonstrate the superiority of the SS-CNN network and evaluate the interactions between network modules, it is essential to conduct ablation experiments to compare and analyze the performance of the deep neural network before and after improvement. Ablation experiments are commonly used with complex neural networks as a method to understand network behavior by removing portions of the network and studying its performance, and have been widely employed by many researchers. In this study, the models subjected to ablation research include SS-CNN, SD-CNN, SE-CNN, 3DCNN, and 2DCNN. Specifically, SD-CNN represents the SS-CNN without the SE attention module, SE-CNN represents the SS-CNN without the superpixelwise division module, 3DCNN represents the SS-CNN without the superpixelwise division module, SE attention module, and 2DCNN, while 2DCNN represents the SS-CNN without the superpixelwise division module, SE attention module, and 3DCNN. Additionally, this study conducted 10-fold cross-validation under different modeling strategies (Tr=0.7 and Tr=0.05) to evaluate model performance.

### 2.4 PCA

PCA (Principal Component Analysis) is an unsupervised learning technique that facilitates data visualization through dimensionality reduction and cluster analysis. It offers a means to gain insights into complex multivariate data and finds extensive use in the processing of Hyperspectral Imaging (HSI) data (Amigo et al., 2013; Bro and Smilde, 2014). It transforms the original spectral information variables into a set of new variables, known as principal components (PCs), which are mutually exclusive and non-overlapping, capturing distinct information. By retaining only, the top PCs with the highest contribution to variance, it effectively represents the primary information within the original data. It’s worth noting that the number of PCs to retain depends on the percentage of cumulative variance they account for within the total variance (Guo et al., 2021). This method yields a simplified set of factors, which can be used for exploration and serves as an efficient graphical representation of the data, offering a precise description of the entire extensive spectral dataset.

### 2.5 Vegetation index

In the realm of remote sensing, vegetation indices derived from remote sensing data provide an effective and direct means for qualitative and quantitative mapping of species identification, vegetation coverage, health status, physiological activity, leaf nitrogen content, leaf area, canopy coverage, and structure. Notable examples of these indices include the Normalized difference vegetation index (NDVI), Photochemical reflectance index (PRI), Enhanced vegetation index (EVI), and Plant senescence reflectance index (PSRI), among other empirical and semi-empirical spectral metrics (Maes and Steppe, 2019). NDVI, in particular, stands as the most widely employed indicator for monitoring crop growth, health status, and vegetative greenness (Li et al., 2019). It is calculated as the disparity between near-infrared and red-region reflectance, divided by the sum of near-infrared and red-region reflectance. NDVI demonstrates a strong correlation with vegetation health, green biomass, and vegetation productivity, as shown in Equation 5:

(5) $$\text{NDVI}=\frac{{\text{ρ}}_{\text{NIR}}-{\text{ρ}}_{\text{RED}}}{{\text{ρ}}_{\text{NIR}}+{\text{ρ}}_{\text{RED}}}$$

where ${\text{ρ}}_{\text{NIR}}$ represents the reflectance values in the near-infrared spectral range, while ${\text{ρ}}_{\text{RED}}$ signifies the reflectance values in the red spectral range.

PRI is a photosynthetic index based on narrow-band reflectance, which is expressed as the difference between the reflectance at 531 nm and the reflectance at 570 nm divided by the sum of the reflectance at 531 nm and the reflectance at 570 nm, as shown in Equation 6. It is considered an effective indicator of plant net photosynthetic rate, CO_2_ uptake, and nutrient deficiency. Net photosynthetic rate is a crucial metric for assessing photosynthetic capacity, vegetation productivity, and the overall growth of plants. A higher net photosynthetic rate indicates better leaf structure and functionality. It is noteworthy that the PRI index is particularly associated with the conversion of carotenoids in the xanthophyll cycle, which is essential to prevent excessive light exposure (Gamon et al., 1992).

(6) $$\text{PRI}=\frac{{\text{ρ}}_{531}-{\text{ρ}}_{570}}{{\text{ρ}}_{531}+{\text{ρ}}_{570}}$$

where ${\text{ρ}}_{531}$ represents the reflectance value at the 531nm wavelength band, and ${\text{ρ}}_{570}$ represents the reflectance value at the 570nm wavelength band.

The enhanced vegetation index (EVI) was analyzed as an indicator of vegetation activity. The index is strongly correlated with chlorophyll content and photosynthetic activity (Huete et al., 2002), and is a normalized ratio of the red, near-infrared, and blue spectral reflectance bands. EVI can have values from −1 to +1. The EVI equation is as shown in Equation 7:

(7) $$\text{EVI}=\frac{2.5({\text{ρ}}_{\text{NIR}}-{\text{ρ}}_{\text{RED}})}{{\text{ρ}}_{\text{NIR}}+6{\text{ρ}}_{\text{RED}}-7.5{\text{ρ}}_{\text{BIUE}}+1}$$

where ${\text{ρ}}_{\text{NIR}}$ represents the reflectance values in the near-infrared spectral band, ${\text{ρ}}_{\text{RED}}$ signifies the reflectance values in the red spectral band, and ${\text{ρ}}_{\text{BIUE}}$ denotes the reflectance values in the blue spectral band.

### 2.6 Classification model evaluation (OA, AA, Kappa)

In this study, we used the overall accuracy (OA), average accuracy (AA), Kappa coefficient, producer’s accuracy (PA) and user’s accuracy (UA) to judge the discriminant effect of deep neural network classification algorithm on rice and weed samples. OA represents the number of correctly classified samples in the overall test sample, which is usually used to evaluate the overall performance of the model (Zhang et al., 2019). AA represents the average accuracy, and Kappa is an index used to measure classification accuracy or consistency, which can reflect the real degree of consistency of classification results. PA represents the probability that the category is correctly classified, while UA represents the probability that the classifier correctly classifies the samples belonging to a particular category (Cao et al., 2018). The closer OA, AA, and Kappa values are to 1, the better the algorithm’s classification performance. The formulas for OA, AA, Kappa, PA, and UA are provided in Equations 8–12:

(8) $$\text{OA}=\frac{\sum_{\text{i}=1}^{\text{n}}{\text{x}}_{\text{ii}}}{\sum_{\text{i}=1}^{\text{n}}\sum_{\text{j}=1}^{\text{n}}{\text{x}}_{\text{ij}}}$$

(9) $$\text{AA}=\frac{1}{\text{n}}\sum_{\text{i}=1}^{\text{n}}\frac{{\text{x}}_{\text{ii}}}{\sum_{\text{j}=1}^{\text{n}}{\text{x}}_{\text{ij}}}$$

(10) $$\text{Kappa}=\frac{\underset{\;\;\;\;\;\;\;\;\;\;\;\;\;\;\;\;\;\;\;\;\text{i}=\text{j}}{\sum_{\text{i}=1}^{\text{n}}\sum_{\text{j}=1}^{\text{n}}{\text{x}}_{\text{ij}}\times \sum_{\text{i}=1}^{\text{n}}{\text{x}}_{\text{ii}}-\sum_{\text{i}=1}^{\text{n}}\left(\sum_{\text{i}=1}^{\text{n}}{\text{x}}_{\text{ij}}\times \sum_{\text{j}=1}^{\text{n}}{\text{x}}_{\text{ij}}\right)}}{\underset{\;\;\;\;\text{i}=\text{j}}{{\left(\sum_{\text{i}=1}^{\text{n}}\sum_{\text{j}=1}^{\text{n}}{\text{x}}_{\text{ij}}\right)}^{2}-\sum_{\text{i}=1}^{\text{n}}\left(\sum_{\text{i}=1}^{\text{n}}{\text{x}}_{\text{ij}}\times \sum_{\text{j}=1}^{\text{n}}{\text{x}}_{\text{ij}}\right)}}$$

(11) $$\text{PA}=\frac{{\text{x}}_{\text{ii}}}{\sum_{\text{j}=1}^{\text{n}}{\text{x}}_{\text{ij}}}$$

(12) $$\text{UA}=\frac{{\text{x}}_{\text{jj}}}{\sum_{\text{i}=1}^{\text{n}}{\text{x}}_{\text{ij}}}$$

where i=1,2,3,⋯,n, represents the number of categories of the real samples; j=1,2,3,⋯,n, represents the number of categories of the predicted samples; n represents the total number of categories; ${\text{x}}_{\text{ij}}$ represents the true number of samples of class i, but the predicted number of samples of class j; ${\text{x}}_{\text{ii}}$ represents the number of samples with both true and predicted i class.

## 3 Results and discussion

### 3.1 Analysis of spectral characteristics of samples

**Figure 6:**
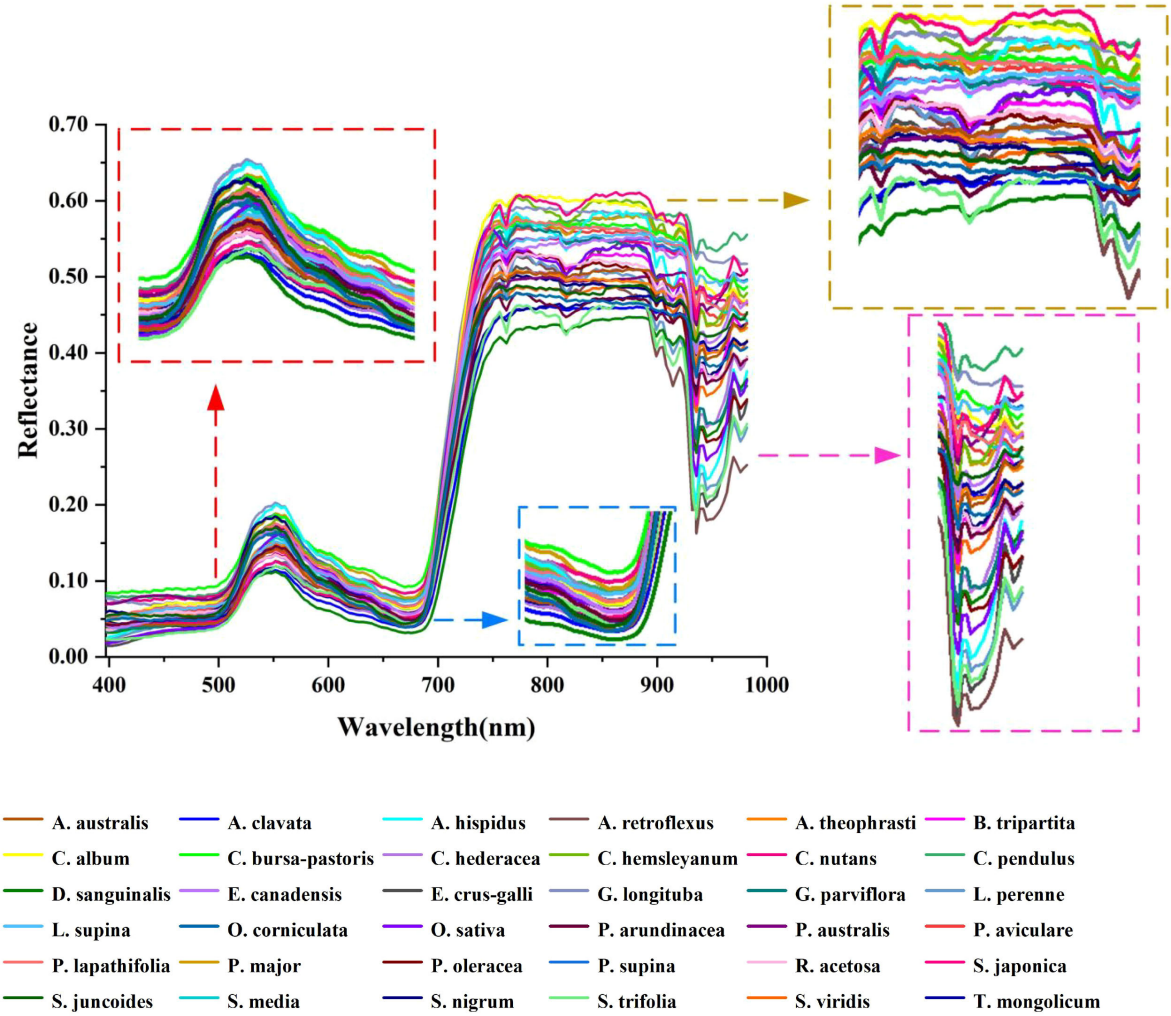
This is a figure. Schemes follow the same formatting.

There is a high noise spectral region (982–1003 nm) in the image acquisition process of rice and weed samples. Consequently, after excluding this region, the spectral acquisition range extracted from the hyperspectral images was 397–982 nm (consisting of 197 wavebands). The reflectance spectra of rice and weed samples are depicted in **Figure 6**. In general, plant spectra will mainly be affected by leaf pigment content in the visible region (400–700 nm) (Yoder and Pettigrew-Crosby, 1995), while the near infrared (NIR) region (700–1100 nm) is highly influenced by leaf and canopy structure that can be affected by phenology as well as species (Gausman, 1985). Rice (*Oryza sativa*, O. sativa) and weed samples exhibit a ‘green peak’ at 550 nm in the visible green region (520–600 nm). This phenomenon occurs because rice and weeds exhibit low photosynthetic activity in this wavelength range, leading to reduced light absorption and consequently higher reflectance (Feret et al., 2008). In the visible red region (630–690 nm), a ‘red valley’ is observed at 680 nm. This spectral band represents the region with the highest chlorophyll absorption and photosynthetic activity in plants, resulting in increased light absorption and decreased reflectance (Herrmann et al., 2011). In the near-infrared region (700–930 nm), the hyperspectral curve rises rapidly, and the curve basically rises to the highest point at 760 nm, forming a reflection platform (Shapira et al., 2013). Near-infrared reflectance is influenced by internal light scattering in leaves, with scattering dependent on anatomical characteristics such as leaf thickness, density, stomatal structure, and so on (Cavender-Bares et al., 2020). A dicotyledonous leaf has more air spaces among its spongy mesophyll tissue than a monocotyledonous leaf (Raven et al., 2005) of the same thickness and age, resulting in a higher reflectance in the NIR region (Gausman, 1985).

In terms of details, several downward reflection valleys are observable, reflecting the pronounced spectral characteristics depicted in **Figure 6**. The reflectance band at 760 nm may be linked to the third overtone O-H stretching of water within plant leaves. Furthermore, a decline in the photosynthetic process could also be indicated by reducing fluorescence energy around 760–810 nm wavelength. When absorbed by chlorophyll, solar energy is used for carbon fixation and heat dissipation, followed by the release of the emission source at longer wavelengths in the form of chlorophyll fluorescence (Krause and Weis, 1991). Furthermore, at 935 nm in the original spectral curve, an absorption trough emerges. At this point, the sharp decrease in spectral reflectance beyond 935 nm suggests that reflectance in the wavelength region exceeding 935 nm is no longer influenced by the leaf’s intrinsic structure. It is well-established that higher water content in plants results in lower spectral reflectance in the NIR range (780 nm-1300 nm) (Zhang et al., 2022b). The decrease in spectral reflectance around the 935 nm wavelength is inferred to be a consequence of cellular fluids, absorbed water, and carbon dioxide emissions within the leaf, as well as the structural properties of cell membranes (Qu et al., 2017).

### 3.2 PCA preliminary analysis

**Figure 7:**
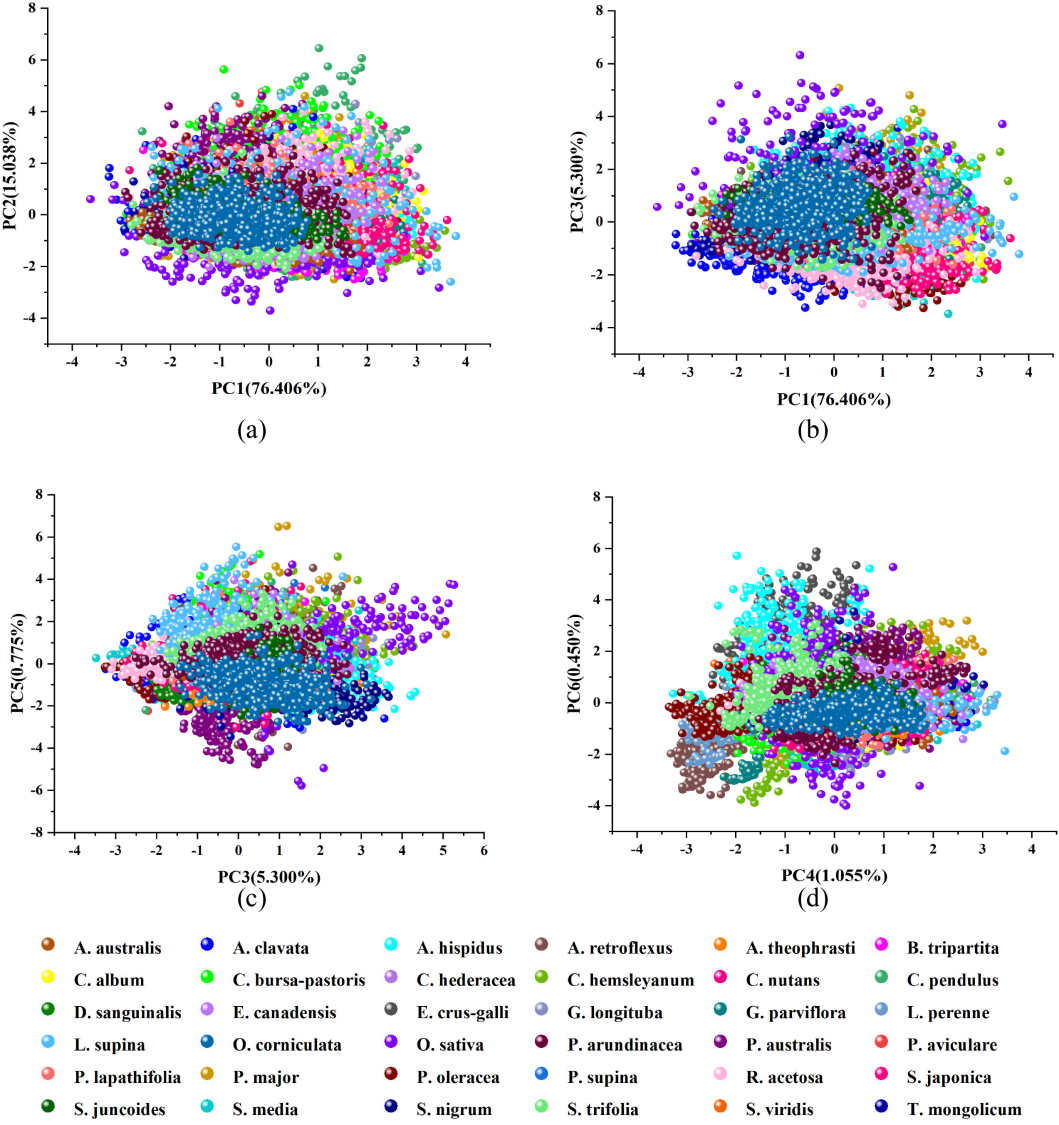
PCA analysis diagram of rice and weed samples. **(a)** PC1-PC2; **(b)** PC1-PC3; **(c)** PC3-PC5; **(d)** PC4-PC6.

PCA was applied to explore the spectral differences and examine the natural pattern among samples in more detail. As illustrated in the PCA analysis graph in **Figure 7**, the first 6 principal components accounted for 99.024% of the total spectral data variance, revealing a complex clustering scenario. Specifically, PC1, PC2, PC3, PC4, PC5, and PC6 contributed to 76.406%, 15.038%, 5.300%, 1.055%, 0.775%, and 0.450% of the variance, respectively. In the PC1-PC2 analysis (**Figure 7a**), rice (*O. sativa*) and weed samples such as *O. corniculata* and *C. pendulus* showed a certain cluster scenario, but the sample clusters of other weeds were very close and not well separated. The PC1-PC3 analysis (**Figure 7b**) demonstrated clearer separability for *A. clavata*, *S. japonica*, and *O. corniculata*. In the PC3-PC5 analysis (**Figure 7c**), species like *L. supina*, *A. clavata*, and *R. acetosa* predominantly occupied the negative side of PC3, *O. sativa*, *S. nigrum*, and *A. hispidus* samples were primarily located on the positive side of PC3. In the PC4-PC6 analysis (**Figure 7d**), distinct clustering and separation patterns were observed among *A. hispidus*, *P. arundinacea*, and *S. media* weed species. A. hispidus was predominantly situated on the positive side of PC6, *P. arundinacea* was located in proximity to the origin of PC6, and *S. media* was primarily distributed on the negative side of PC6. Based on the aforementioned PCA analysis results, it becomes evident that the clustering of most weed samples exhibits a high degree of overlap, weakening their separability. This may be attributed to the fact that rice and various weed species are all green plants with similar spectral fingerprints. In summary, while PCA analysis does not provide precise differentiation among sample types, it does reveal clustering and separation patterns between rice and weed samples. Therefore, further differentiation can be achieved using end-to-end deep learning modeling.

### 3.3 Vegetation index analysis

**Figure 8:**
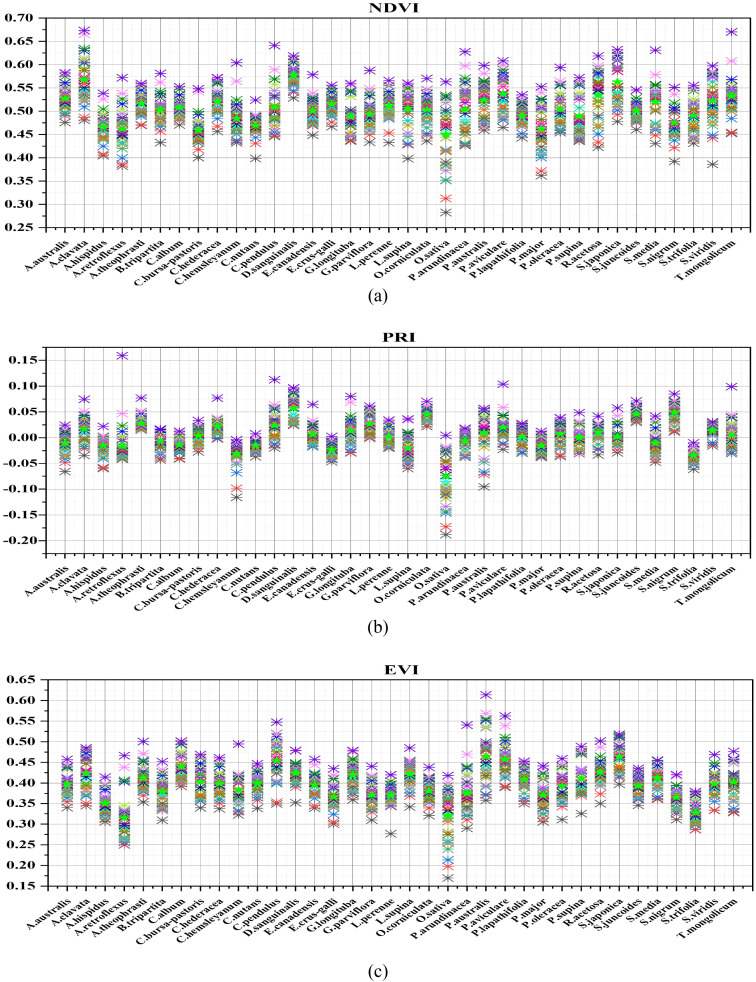
Vegetation index distribution map of rice weed samples (**a** NDVI, **b** PRI, **c** EVI), ★ represents the average value of vegetation index for each sample, ✱ represents the average vegetation index value of the sample on each hyperspectral image of a species (each hyperspectral image is represented by different colors).

The high spectral resolution (more narrowband) images obtained by the SPECIM IQ provide ample spectral information, particularly in the visible and near-infrared spectral wavelength regions. These narrow bands can be effectively employed for the study of various weed species with different growth and physiological conditions in the Northern Chinese paddy field environment. **Figure 8** illustrates the variations of NDVI, PRI, and EVI indices among different weed species. In **Figure 8a**), the NDVI levels reflect the levels of plant growth and physiological condition, indicating that the growth of various weeds is not the same (Bai et al., 2019). Samples of *D. sanguinalis*, *A. clavata*, and *S. japonica* exhibit higher NDVI values overall, while O. sativa samples display lower overall NDVI values. *P. oleraceum*, *B. tripartita*, and *P. arundinacea* exhibit intermediate NDVI values. The underlying reasons for this phenomenon likely stem from differences in the growth environments of rice and weed species, as well as the inherent physiological structure and activity of each species. Furthermore, the NDVI values of *A. australis*, *P. australis*, *R. acetosa*, *S. viridis*, and *T. mongolicum* tend to be consistent, indicating that these weed species share similar growth status.

In the realm of plant vegetation indices, another crucial metric is PRI (Photochemical Reflectance Index), which is associated with the plant’s xanthophyll cycle and the photosynthetic light use efficiency (Fréchette et al., 2016). **Figure 8B** depicts the distribution of PRI results among various samples. In comparison to the distribution of NDVI, the distribution of PRI of each sample species changed. *D. sanguinalis*, *S. nigrum*, and *S. juncoides* display the highest PRI values. *T. mongolicum*, *C. bursa-pastoris*, and *P. lapathifolia* fall within the intermediate range of PRI values. Conversely, PRI values are lowest for *C. hemsleyanum*, *E. crus-galli*, *O. sativa*, and *S. trifolia*. This suggests variations in xanthophyll content levels and the efficiency of photosynthetic light utilization among different rice and weed species. The underlying reasons may be linked to the synthesis of xanthophylls within chloroplasts, which is related to the photosynthetic process of plants. Nevertheless, inherent physiological traits of plants, as well as disparities in photosynthetic light use efficiency, contribute to differing levels of photosynthesis, resulting in distinct PRI distributions among plant species. It’s worth noting that, in contrast to the distribution of NDVI values, the distinction among PRI values for the samples is somewhat reduced. Notably, *O. sativa* exhibits the lowest PRI value and displays a substantial PRI difference compared to other weed species, indicating its lower xanthophyll content.

**Figure 8C** illustrates the distribution of EVI values for various samples. *P. australis*, *S. japonica*, and *P. aviculare* samples exhibit higher EVI values, while *O. sativa* and *A. retroflexus* samples generally show lower EVI values. Additionally, *C. nutans*, *C. hederacea*, and *S. viridis* fall in the middle range of EVI values. This phenomenon can be attributed to the relationship between EVI and the internal chlorophyll content and photosynthetic activity of the plants. Chlorophyll content, a key component of photosynthesis, significantly influences light absorption and scattering. With increasing chlorophyll content and photosynthetic activity, the photosynthetic process of the plants is enhanced. Notably, *P. australis* exhibits the highest EVI values, likely due to its unique physiological structure, resulting in the highest chlorophyll content and photosynthetic activity. The analysis of NDVI, PRI, and EVI results reveals variations in vegetation indices among individual samples due to differences in plant growth conditions, physiological activities, and environmental factors. However, the vegetation index distributions for most samples are similar, lacking significant differentiation. Therefore, it is essential to further differentiate different species samples using deep learning models.

### 3.4 Classification and visualization results under normal training samples (Tr = 0.7)

**Figure 9:**
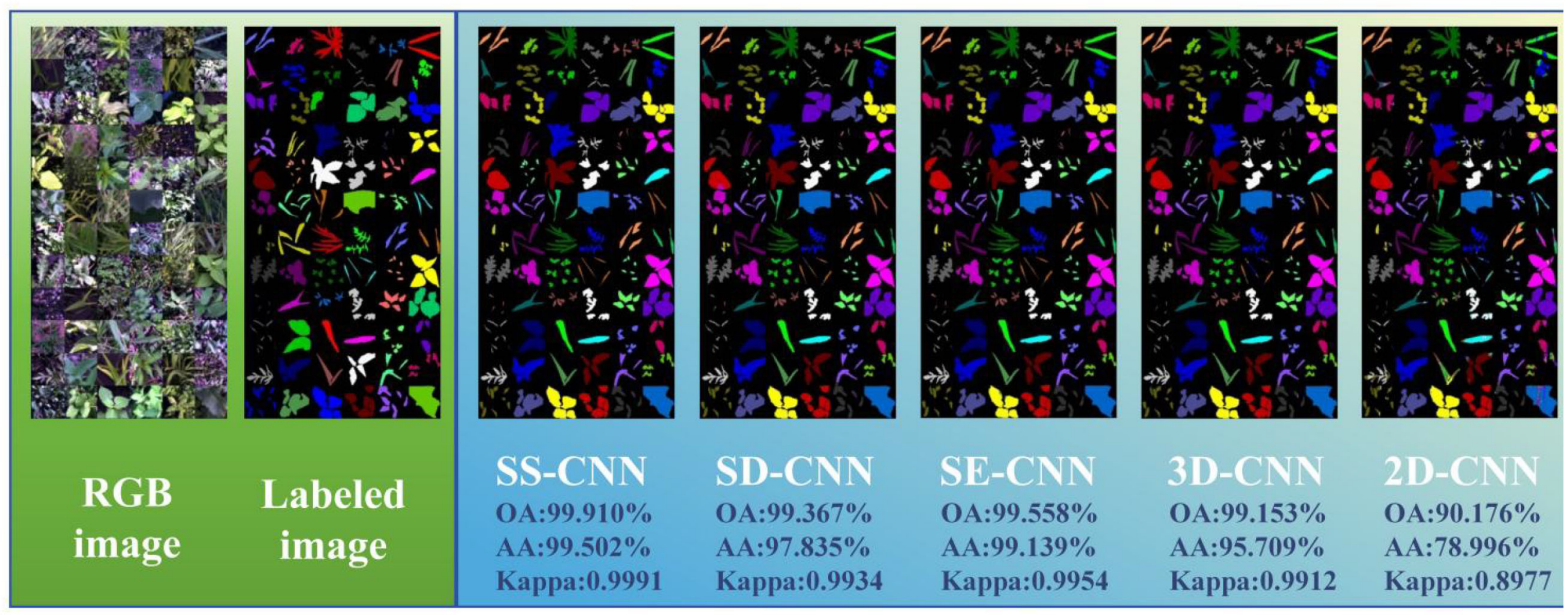
The classification and visualization results of each classification model under normal training samples (Tr =0.7) (Label image: 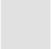
A. australis, 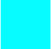
A. clavata, 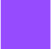
A. hispidus, 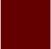
A. retroflexus, 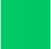
A. theophrasti, 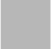
B. tripartita, 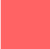
C. album, 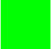
C. bursa-pastoris, 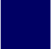
C. hederacea, 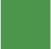
C. hemsleyanum, 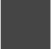
C. nutans, 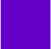
C. pendulus, 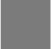
D. sanguinalis, 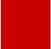
E. canadensis, 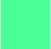
E. crus-galli, 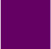
G. longituba, 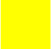
G. parviflora, 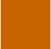
L. perenne, 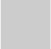
L. supina, 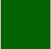
O. corniculata, 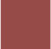
O. sativa, 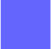
P. arundinacea, 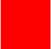
P. australis, 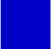
P. aviculare, 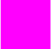
P. lapathifolia, 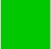
P. major, 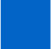
P. oleracea, 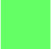
P. supina, 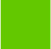
R. acetosa, 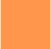
S. japonica, 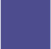
S. juncoides, 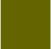
S. media, 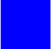
S. nigrum, 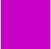
S. trifolia, 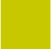
S. viridis, 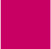
T. mongolicum9).

In this study, discrimination analysis of rice and weed samples was performed using various classification algorithms, including SS-CNN, SD-CNN, SE-CNN, 3DCNN, and 2DCNN. According to the discriminant effect of the model, the optimal model suitable for the classification of rice and weed samples was obtained. **Figure 9** illustrates the classification results and visualizations of SS-CNN, SD-CNN, SE-CNN, 3DCNN, and 2DCNN algorithms on the test dataset. The results from the discrimination analysis reveal that the classification algorithm constructed by SS-CNN outperforms other classification algorithms in terms of OA, AA, and Kappa values, signifying a significant improvement in modeling accuracy. Notably, compared to the 2D-CNN algorithm, SS-CNN exhibits a more substantial enhancement in modeling accuracy. Specifically, the OA value of the SS-CNN algorithm is 0.543% higher than that of SD-CNN, 0.352% higher than SE-CNN, 0.757% higher than 3D-CNN, and 9.734% higher than 2D-CNN (**Table 1**). The AA value increases by 1.667%, 0.363%, 3.793%, and 20.506% in the same order, while the Kappa value increases by 0.0057, 0.0037, 0.0079, and 0.1014. Furthermore, the results of PA and UA for the optimal SS-CNN algorithm (**Table 1**) demonstrate that the accuracy is highest for species such as *A. australis*, *B. tripartita*, *C. album*, *C. hemsleyanum*, *C. nutans*, *E. crus-galli*, *G. longituba*, *L. perenne*, *L. supina*, *O. corniculata*, *O. sativa*, *P. aviculare*, *P. lapathifolia*, *P. supina*, *S. japonica*, *S. juncoides*, *S. media*, *S. trifolia*, and *T. mongolicum*, all reaching a PA and UA value of 100%. In contrast, species like *A. clavata*, *G. parviflora*, and *S. nigrum* exhibit relatively lower accuracy, with UA values of 87.879%, 98.561%, and 97.600%, respectively. The reason for this result may be that *A. clavata*, *G. parviflora*, *S. nigrum* have no obvious distinguishable morphological features and exhibit high similarities to samples of other species. When classifying samples, they are misclassified as other species to a greater extent, resulting in a smaller UA value.

As shown in **Figure 9**, the discrimination results of rice and weed species across different deep neural network classification models are visualized, and corresponding color labeling is applied to samples of different species. From the original RGB images, it is evident that both rice and weed samples generally appear green in color. Apart from a few samples with distinct visual characteristics, the majority of samples cannot be easily distinguished by the naked eye. Comparing the image recognition results of different classification models, the SS-CNN algorithm achieved excellent detection performance, with an OA of 99.910% on the test set, correctly labeling almost all samples. In contrast, the 2D-CNN algorithm demonstrated relatively poor detection performance, with an OA of 90.176% on the test set, showing mislabeling among different sample types. This phenomenon may be attributed to the fact that 2D-CNN primarily focuses on local features when extracting image information, providing limited grasp of the global image information, thus leading to reduced image classification accuracy. However, for the classification results under Tr=0.7, the detection performance of the optimized classification algorithms (SS-CNN, SD-CNN, SE-CNN) after superpixel segmentation and SE attention mechanism modules outperformed 3D-CNN and 2D-CNN. Importantly, the proposed SS-CNN in this study achieved a classification accuracy of 99.910%, exhibiting high consistency with the ground truth images. Consequently, classifying rice and weed using the SS-CNN algorithm is deemed feasible.

### 3.5 The classification and visualization results under few-shot learning (Tr =0.05)

**Figure 10:**
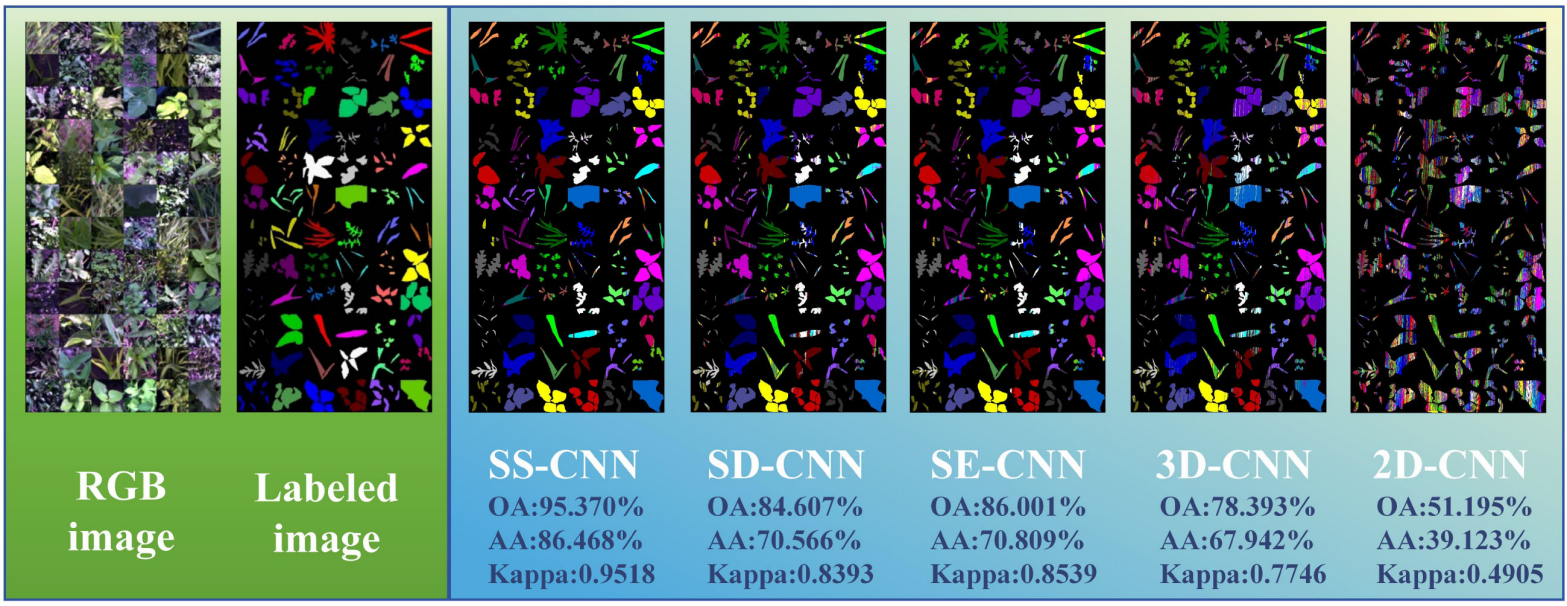
The classification and visualization results of each classification model in the case of few-shot learning (Tr =0.05) (Label image: 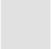
A. australis, 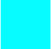
A. clavata, 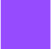
A. hispidus, 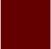
A. retroflexus, 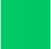
A. theophrasti, 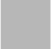
B. tripartita, 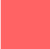
C. album, 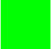
C. bursa-pastoris, 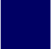
C. hederacea, 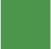
C. hemsleyanum, 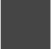
C. nutans, 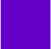
C. pendulus, 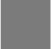
D. sanguinalis, 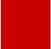
E. canadensis, 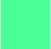
E. crus-galli, 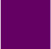
G. longituba, 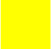
G. parviflora, 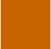
L. perenne, 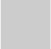
L. supina, 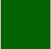
O. corniculata, 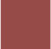
O. sativa, 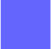
P. arundinacea, 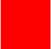
P. australis, 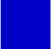
P. aviculare, 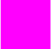
P. lapathifolia, 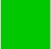
P. major, 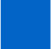
P. oleracea, 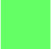
P. supina, 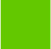
R. acetosa, 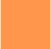
S. japonica, 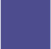
S. juncoides, 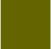
S. media, 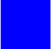
S. nigrum, 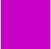
S. trifolia, 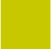
S. viridis, 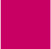
T. mongolicum).

Deep learning algorithms has been widely used in hyperspectral image classification tasks and have achieved favorable classification results. As long as there is available data, it can represent the changes in practice. However, in the field of agriculture, the available data for building robust models is severely limited due to challenging field conditions, extended experiment periods and budget constraints. To address this, as depicted in **Figure 10**, we explore the classification outcomes of deep learning algorithms in the case of few-shot learning, where the training dataset accounts for only 5% of the total samples. Among these algorithms, the SS-CNN algorithm achieves the highest classification accuracy for rice and weeds, with OA, AA, and Kappa values of 95.370%, 86.468%, and 0.9518, respectively. Compared to the SD-CNN, SE-CNN, 3D-CNN, and 2D-CNN algorithms, the SS-CNN algorithm exhibits successive improvements in OA by 10.763%, 9.369%, 16.977%, and 44.175%, AA by 15.902%, 15.659%, 18.526%, and 47.345%, and Kappa by 0.1125, 0.0979, 0.1772, and 0.4613, respectively. From the classification results of deep neural network algorithms, it can be seen that in the case of Tr=0.05 training samples, the classification accuracy of each algorithm is lower than that of Tr=0.7 training samples. Notably, for SS-CNN, its OA declines by 4.540% in the case of Tr=0.05 training samples but still achieves satisfactory results. This underscores the robust applicability of SS-CNN even in scenarios with limited training samples.

Specifically, the results of PA and UA for SS-CNN are presented in **Table 2**, demonstrating high accuracy for the following plant species: *A. australis*, *A. retroflexus*, *C. bursa-pastoris*, *C. nutans*, *C. pendulus*, *E. crus-galli*, *G. longituba*, *L. perenne*, *L. supina*, *O. sativa*, *P. supina*, *S. japonica*, and *S. juncoides*, with both PA and UA values exceeding 95%. On the other hand, the accuracy for *A. clavata*, *B. tripartita*, and *P. major* species is relatively lower, with *A. clavata* having PA and UA values of 77.328% and 62.584%, *B. tripartita* with PA and UA values of 88.046% and 85.373%, and *P. major* with PA and UA values of 81.006% and 43.873%. Compared to the results of normal training samples, the accuracy of species such as *A. australis*, *A. clavata*, *B. tripartita*, *C. nutans*, *E. crus-galli*, *G. longituba*, *L. perenne*, *L. supina*, *O. sativa*, *P. supina*, *S. japonica*, and *S. juncoides* have decreased. Notably, *A. clavata* exhibited the most significant reduction in accuracy, with PA and UA values decreasing from 100% and 87.879% to 77.328% and 62.584%. The reason behind this phenomenon may be attributed to the relatively small proportion of pixel points in the original hyperspectral images of *A. clavata*. During deep learning algorithm training with a limited sample size, it may fail to capture these pixel points, resulting in the decline of PA and UA values.

**Table 2:** The recognition results of rice and weeds by the optimal algorithm SS-CNN under different training ratio conditions.

	Tr=0.7		Tr=0.05	
	PA/%	UA/%	PA/%	UA/%
A. australis	100.000	100.000	96.750	98.902
A. clavata	100.000	87.879	77.328	72.584
A. hispidus	99.725	100.000	84.687	94.644
A. retroflexus	99.927	100.000	96.011	99.983
A. theophrasti	100.000	99.845	94.441	99.270
B. tripartita	100.000	100.000	88.046	85.373
C. album	100.000	100.000	94.475	96.188
C. bursa-pastoris	99.904	100.000	99.390	99.955
C. hederacea	100.000	99.740	90.646	92.545
C. hemsleyanum	100.000	100.000	94.927	85.482
C. nutans	100.000	100.000	96.936	96.891
C. pendulus	99.901	100.000	97.582	98.823
D. sanguinalis	99.833	100.000	90.749	99.921
E. canadensis	99.779	100.000	95.723	94.773
E. crus-galli	100.000	100.000	97.557	98.764
G. longituba	100.000	100.000	95.691	97.169
G. parviflora	97.857	98.561	94.488	20.339
L. perenne	100.000	100.000	98.821	100.000
L. supina	100.000	100.000	98.540	99.826
O. corniculata	100.000	100.000	97.980	87.717
O. sativa	100.000	100.000	97.708	98.829
P. arundinacea	99.289	100.000	84.290	94.363
P. australis	99.225	99.482	91.667	88.135
P. aviculare	100.000	100.000	91.820	95.031
P. lapathifolia	100.000	100.000	91.728	80.441
P. major	99.363	100.000	81.006	43.873
P. oleracea	99.805	99.418	94.684	99.725
P. supina	100.000	100.000	98.264	100.000
R. acetosa	100.000	99.885	92.308	97.535
S. japonica	100.000	100.000	97.631	99.329
S. juncoides	100.000	100.000	96.117	96.954
S. media	100.000	100.000	99.182	82.668
S. nigrum	99.187	97.600	100.000	11.509
S. trifolia	100.000	100.000	90.526	95.771
S. viridis	100.000	99.671	95.858	90.039
T. mongolicum	100.000	100.000	89.143	92.067

The visualization results at Tr = 0.05 show the change of classification results of deep neural networks under different modeling strategies. Both 3D-CNN and 2D-CNN algorithms exhibit issues related to misclassification among diverse weed species. This phenomenon can be attributed not only to the limited global information comprehension of the entire image by the 3D-CNN and 2D-CNN algorithms but also to the challenge of these models in learning and capturing the underlying non-linear relationships within the rice and weed sample data as the labeled samples decrease. Similarly, consistent with the modeling strategy of Tr =0.7, in the case of Tr =0.05, the classification algorithm (SS-CNN, SD-CNN, SE-CNN) optimized by the superpixelwise division module and the SE attention mechanism module is superior to the detection effect of 3D-CNN and 2D-CNN. Notably, the SS-CNN algorithm proposed in this study achieved a classification accuracy of 95.370%, with recognition performance highly consistent with labeled images.

### 3.6 Discussion

In recent years, the increase in summer temperatures and precipitation in northern China has created favorable conditions for the frequent occurrence of plant diseases, pests, and species invasions. Consequently, the establishment of a spectral library for northern Chinese paddy field weeds and weed stress species identification monitoring has become increasingly crucial. Previous studies have primarily focused on analyzing the spatial distribution and species identification of weeds in farmlands using large-scale remote sensing data and multispectral data from drones. Some studies have also assessed the impact of competitive pressure on ecological weed management (Osorio et al., 2020; Reedha et al., 2022). However, weed stress identification and monitoring at the field scale pose significant challenges, as applications such as precision agriculture and targeted spraying require more detailed information about weed species and physiological activities than ever before (Li et al., 2023). Therefore, on-site and laboratory-based hyperspectral analysis, especially in the challenging field environments, holds immense potential in the characterization and discriminative analysis of weed stress, thanks to its high resolution. Based on this premise, our research utilizes hyperspectral imaging technology to establish a hyperspectral library of rice and weed species in the cold regions of northern China. Compared to the study by Dmitriev et al (Dmitriev et al., 2022), which examined five weed species, our research benefits from a greater diversity of weed species, broader geographical coverage, and a larger volume of spectral data. Canopy spectral profile characteristics, vegetation indices, and principal component analysis (PCA) explain the differences in physiological activities of different weed species and the inherent distribution patterns of spectral data. The wavelengths around 550 nm, 680 nm, 760 nm, and 935 nm, as well as their neighboring spectral bands, are considered to represent the most prominent spectral features of weeds. They reflect the spectral diversity of weed species and indirectly indicate their physiological activities, potentially playing a crucial role in weed identification. The results of Bai et al (Bai et al., 2013). showed that there were obvious reflection peaks in the spectral curves of all weeds in the wavelength range of 508–576 nm, and the reflectance of the spectral curves in the wavelength range of 682–739 nm increased rapidly, and the reflectance curves of all weeds in the wavelength range of 780–1000 nm were significantly different, which was similar to the results of this study. In addition to the species and physiological activity of weeds, the spectral characteristics of weeds may also vary under different environmental conditions. Within agricultural ecosystems, distinct soil and climate conditions can lead to variations in weed species and their growth patterns (Carlesi et al., 2013; Steponavičienė et al., 2021). For example, the study of Henry et al (Henry et al., 2004). shows that the characteristics of Discrete Wavelet Transformation (DWT) spectral reflectance curves of weed (*Xanthium strumarium L.* and *Cassia obtusifolia L.*) are greatly affected by different soil moisture, and the trend of their spectral curves in the visible region (400-780nm) and the near-infrared (NIR) region (780-2400nm) differ significantly from the original curve.

Moreover, research conducted by Zhang and Slaughter (2011) explored the impact of environmental temperature changes on the hyperspectral imaging characteristics of weeds in the visible and near-infrared regions. Increased temperature leads to higher spectral reflectance in the visible range (480-670nm) of weed canopies, while, during the same conditions, reflectance in the range of 720-810nm decreases. Additionally, vegetation indices may vary with the growth stages of weeds. A vegetation index shows statistical differences in different weed phenological stages and provides a valuable reference for the differentiation of weeds in different growth periods (Peña-Barragán et al., 2006). However, due to the limitation of conditions, the current research did not consider the physiological activity of various weeds in different growth environments and growth periods, as well as the biochemical and physiological responses of rice under different growth conditions and their spectral characteristics. Future studies should aim to bridge the gap between sensitive spectral features and the potential biochemical and physiological processes that rice undergoes throughout its entire growth cycle in response to various modes of weed stress.

In the discrimination analysis of rice and weed stress, this study established a deep learning network, termed SS-CNN, and conducted ablation experiments. Under the condition of Tr=0.7, the SS-CNN model outperformed the comparative models in terms of recognition (OA: 99.910%, AA: 99.502%, Kappa: 0.9991). Similarly, at Tr=0.05, the SS-CNN classification algorithm still achieved the best classification results (OA: 95.370%, AA: 86.468%, Kappa: 0.9518). The ablation experiments investigated the impact of the superpixelwise division (SD) module and SE Attention module on the detection performance of the SS-CNN algorithm. The classification results under Tr=0.7 showed an improved detection performance of the SS-CNN algorithm with both SD and SE modules compared to the SD-CNN algorithm (OA: 99.367%) and the SE-CNN algorithm (OA: 99.558%). It also outperformed the 3D-CNN (OA: 99.153%) and the 2D-CNN (OA: 90.176%) to a greater extent. The detection accuracy of the SS-CNN was 0.543% higher than that of the SD-CNN, demonstrating the SE Attention module’s enhancement of the deep learning algorithm for discriminating between rice and weed samples. Furthermore, the detection accuracy of the SS-CNN was 0.352% higher than that of the SE-CNN, confirming the effectiveness of the superpixelwise division module in improving the deep learning algorithm’s detection performance. Especially in the case of Tr=0.05, the detection accuracy of the SS-CNN significantly exceeded that of the SD-CNN and SE-CNN, underscoring the positive role of the SE attention and superpixelwise division modules in deep learning algorithms. These findings further highlight the superior performance of the SS-CNN.

In comparison to the most advanced existing research, for example, Xu et al (Xu et al., 2020). proposed a natural weed identification method that integrates RGB image features and deep features. This method extracts color, positional, textural, and depth features from RGB images and depth images during the tillering and jointing stages of crops. The AdaBoost algorithm was then used to identify weeds, achieving an accuracy of 88% at the tillering stage and 81.08% at the jointing stage. Zhang et al (Zhang et al., 2022a). proposed an EM-YOLOv4-Tiny weed recognition model based on machine vision technology, which combines multi-scale detection and attention mechanism. This model was used to identify six weed species in peanut fields (*Portulaca oleracea L.*, *Eleusine indica (L.)*, *Chenopodium album L.*, *Amaranthus blitum L.*, *Abutilon theophrasti Medicus* and *Calystegia hederacea Wall. ex Roxb*), achieving a Mean Average Precision (mAP) of 94.54% on the test dataset. The SS-CNN model still achieved the best results, and it is worth mentioning that the weed species targeted in this study are the most. This suggests that the SS-CNN model’s effectiveness in distinguishing various weeds from rice plants remains robust even in challenging field conditions. Furthermore, the utility of such predictions can be used in the area of global food security, especially in the detection of rice weed stress.

Compared with the satisfactory accuracy obtained by the SS-CNN model in hyperspectral, similar studies have used multi-spectral aerial photos to detect rice weeds (Yu et al., 2022). Hyperspectral technology provides better accuracy for rice weed identification, because a large number of studies have shown that the mapping accuracy of plants is improved with higher spectral resolution (Ferreira et al., 2016; Awad, 2018). However, a major limitation of the widespread use of hyperspectral technology for weed species identification is that it is difficult to determine reliable calibration procedures and spectral algorithms in many environments (such as soil type, growth stage, variety, and weather). In practical scenarios, remote sensing alone cannot quantify the relationship between plant spectral characteristics and soil, growth period, weather, variety or management at a specific time and place. Hence, understanding the distinctions between these factors and developing a universal algorithm becomes crucial, which may involve techniques such as physiological analysis, mechanistic models, crowdsourcing, and transfer learning. We aim to formulate objective solutions to improve resource productivity in the future. In addition, the research results will contribute to variable spraying, targeted weed management, and growth monitoring based on weed species specificity.

## 4 Conclusion

This study employed hyperspectral imaging technology to characterize and discriminate between Northern Chinese rice and weeds. In this research, canopy spectral profiles, along with PCA and vegetation indices of rice and weed samples, were utilized for spectral feature analysis and physiological activity characterization, revealing characteristic wavelengths for weeds, including 550 nm, 680 nm, 760 nm, and 935 nm, indicative of species traits. A deep learning algorithm, SS-CNN, was developed, incorporating a superpixel division module and SE attention module, and subjected to ablation experiments under different modeling strategies. The results demonstrated that the SS-CNN classification algorithm performed best on sample identification at Tr=0.7 training (OA: 99.910%, AA: 99.502%, Kappa: 0.9991). Similarly, at Tr=0.05 training, although the identification performance of the SS-CNN classification algorithm declined compared to Tr=0.7, it still achieved optimal classification results (OA: 95.370%, AA: 86.468%, Kappa: 0.9518). The proposed methodology plays a significant role in characterizing and discriminating between rice and weeds in harsh field environments. Furthermore, this study not only enriches the spectral library data for field weed stress but also holds potential value in precise monitoring and management decision-making for field crops, advancing plant phenotyping development.

## Data Availability

The raw data supporting the conclusions of this article will be made available by the authors, without undue reservation.
